# Some coral diseases track climate oscillations in the Caribbean

**DOI:** 10.1038/s41598-017-05763-6

**Published:** 2017-07-18

**Authors:** C. J. Randall, R. van Woesik

**Affiliations:** Department of Biological Sciences, Florida Institute of Technology Melbourne, Florida, United States of America

## Abstract

Disease outbreaks continue to reduce coral populations worldwide. Understanding coral diseases and their relationships with environmental drivers is necessary to forecast disease outbreaks, and to predict future changes in coral populations. Yet, the temporal dynamics of coral diseases are rarely reported. Here we evaluate trends and periodicities in the records of three common coral diseases (white-band disease, yellow-band disease, and dark-spot syndrome) that were surveyed between 1997 and 2014 at 2082 sites throughout the Caribbean. The relationship between the periodicities of disease prevalence and El Niño Southern Oscillation (ENSO) cycles was examined using cross-wavelet analyses and convergent cross mapping (CCM). The prevalence of the diseases peaked every two to four years, and matched periodicities in ENSO conditions. CCM models suggested that environmental conditions associated with recent ENSO cycles may have influenced the patterns in disease prevalence. We also found no increasing trends in disease prevalence through time. Instead, our work suggests that the prevalence of coral diseases is dynamic and complex. The gradual increase in sea-surface temperature, a consequence of increasing greenhouse gas emissions, progressively raises the modal temperature threshold of each ENSO cycle. These dynamic cycles and the increasing modal temperatures appear to influence the dynamics of coral diseases.

## Introduction

Diseases have contributed to the decline of many coral species in the Caribbean, and the numbers of new and emerging diseases are purportedly increasing^1, 2^. Many outbreaks of coral diseases in the Caribbean are associated with periods of elevated sea-surface temperature, which likely cause the tripartite relationship – between the coral host, the pathogen, and the environment – to shift in favor of the pathogen. The most commonly observed and thermally-driven coral diseases in the Caribbean are: (i) white-band disease^3^, (ii) yellow-band disease^4^, and (iii) dark-spot syndrome^5^. The incidence and severity of these coral diseases often have peaked during anomalously high sea-surface temperatures^3, 5, 6^, have followed coral-bleaching events^6–10^, and have led to the development of temperature-based disease surveillance tools^11^. Yet, few studies have evaluated temporal patterns in the prevalence of coral diseases, but see refs 12–14. Consequently, there is little evidence that the prevalence of coral diseases oscillates, or that coral diseases coincide with climatic cycles, such as the El Niño Southern Oscillation (ENSO). Indeed, these short-term climatic cycles may influence the dynamics of Caribbean-coral diseases because the cycles drive fluctuations in sea-surface temperatures^15^ that extend to the North Atlantic and the Caribbean^16^ (Supplementary Figure 12).

Here we examined whether the prevalence of coral diseases fluctuated predictively over the last eighteen years in the Caribbean. We asked whether any fluctuations in disease prevalence coincided with ENSO-driven climate oscillations. We also evaluated whether there were any regional 18-year trends in the prevalence of coral diseases. Data on the prevalence of three prominent coral diseases were obtained between 1997 and 2014 at 2082 sites throughout the Caribbean (http://www.agrra.org/). Three diseases were targeted: (1) white-band disease, (2) yellow-band disease, and (3) dark-spot syndrome. These diseases were selected because they are common and widespread^9^, and because they have all contributed to the significant loss of corals in the Caribbean^6, 7, 9, 17, 18^. Three disease responses were examined, including: (1) the average prevalence of disease, (2) the maximum prevalence of disease, and (3) the variance in the prevalence of disease. We also obtained data for two prominent ENSO indices, the Southern Oscillation Index (SOI), and the Oceanic Niño Index (ONI) (National Oceanic and Atmospheric Administration). We used Morlet wavelet-transform analyses to decompose the signal of each disease and each climate index into time-frequency space, and to examine the timing of significant periodicities. Cross-wavelet analyses were used to determine whether the significant frequencies observed in the coral-disease data corresponded with similar frequencies in the ENSO indices. Convergent cross mapping analyses were used to assess the presence of a causal relationship between the ENSO indices and the disease records. We also used generalized linear models to evaluate whether there were long-term trends in the prevalence of each disease over the 18-year period.

**Figure 1:**
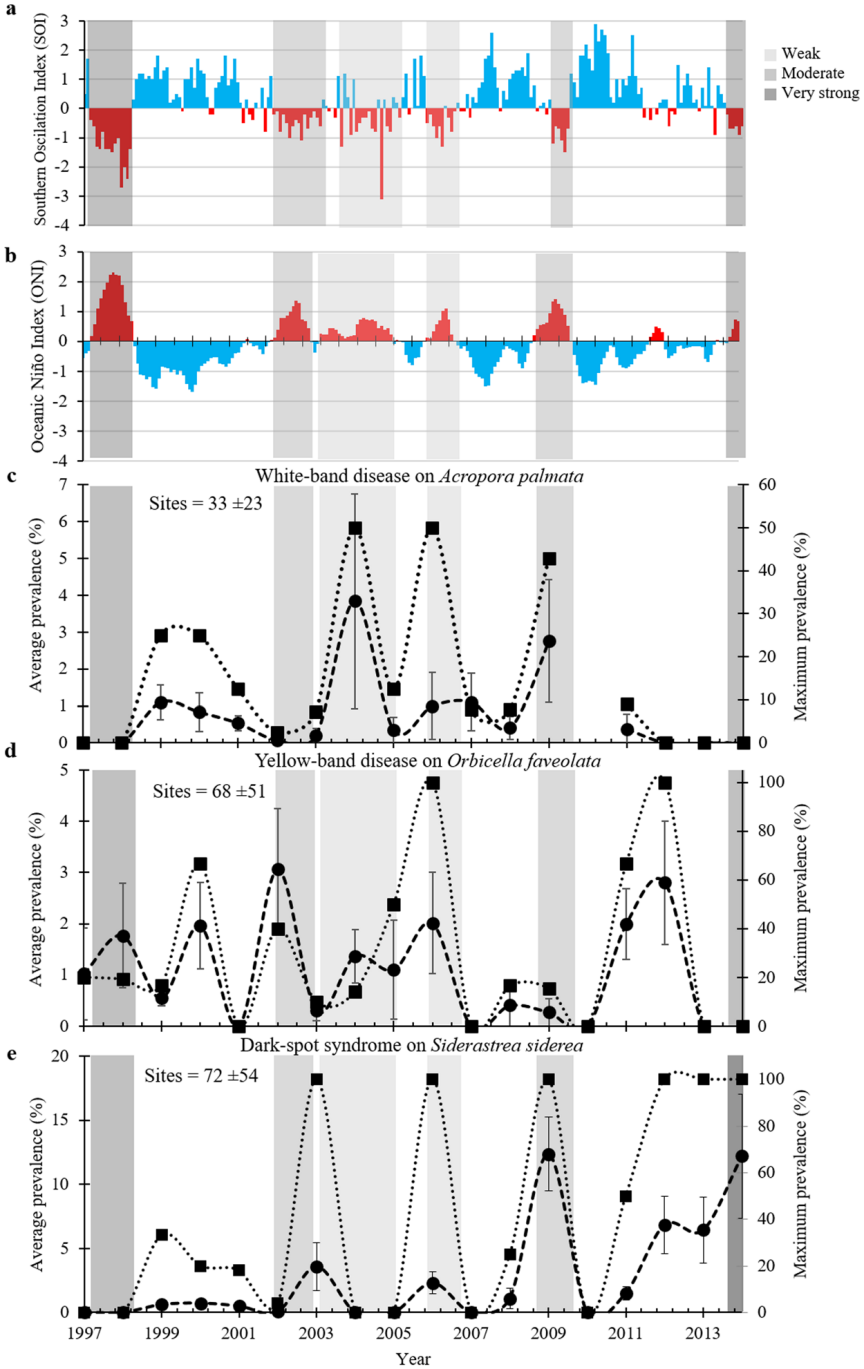
El Niño Southern Oscillation indices and average disease prevalence through time. (**a**,**b**) are both indices of the El Niño Southern Oscillation. (**a**) illustrates the Southern Oscillation Index (SOI), and (**b**) illustrates the Oceanic Niño Index (ONI). Red bars indicate El Niño-like conditions whereas blue bars indicate La Niña-like conditions. (**c**–**e**) indicate average yearly prevalence (%, circles and dashed lines) and maximum yearly prevalence (%, squares and dotted lines) of coral diseases in the Caribbean from 1997–2014. Error bars on the average prevalence indicate ±1 standard error of the mean. Values in the top left of panels of (**c**–**e**) indicate the average yearly sample size for mean prevalence calculations ±1 standard deviation. Shaded areas indicate the strength of the El Niño events: Dark gray indicates very strong El Niño events (1.5–1.9 °C sea-surface temperature (SST) anomaly), gray indicates moderate El Niño events (1.0–1.4 °C SST anomaly), and light gray indicates weak El Niño events (0.5–0.9 °C SST anomaly).

**Figure 2:**
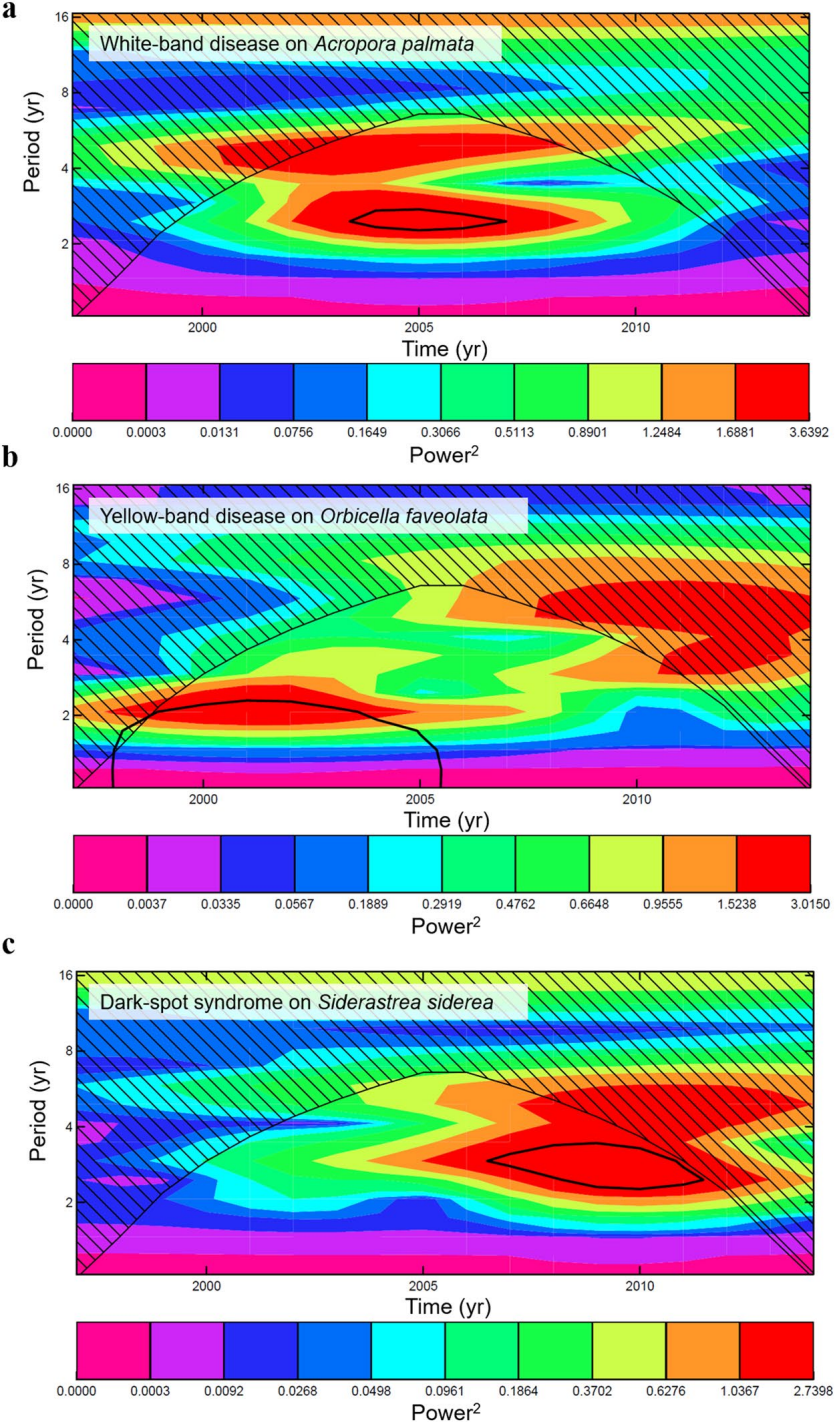
Morlet wavelet-transform analyses identifying periodicities in the prevalence of three coral diseases. Yearly average prevalence of three coral diseases in the Caribbean from 1997–2014 were used in these analyses. Regions within black contour lines indicate statistically significant periodicites at p < 0.05. Dashed areas indicate the region outside the ‘cone of influence’. Color bars indicate the power spectra.

**Figure 3:**
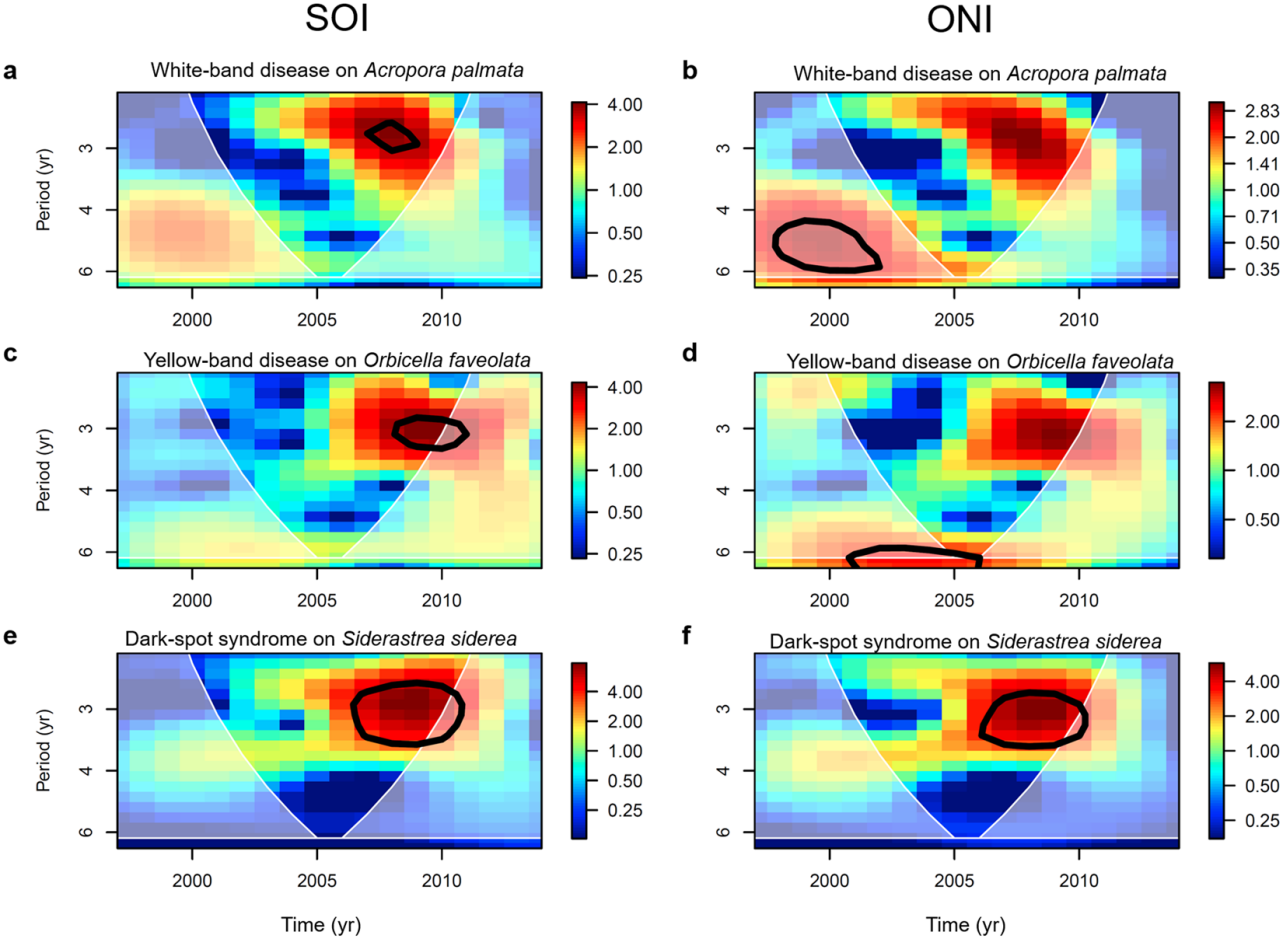
Cross-wavelet analyses comparing ENSO indices and disease records. Cross-wavelet analyses comparing yearly maximum prevalence of three coral diseases with two indices of El Niño Southern Oscillation (ENSO): The Southern Oscillation Index (SOI; left) and the Oceanic Niño Index (ONI; right). ENSO indices were compared with the prevalence of white-band disease on *Acropora palmata* (**a**,**b**), yellow-band disease on *Orbicella faveolata* (**c**,**d**), and dark-spot syndrome on *Siderastrea siderea* (**e**,**f**). Black contour lines indicate statistically significant periodicites at p < 0.05. The white lines outline the ‘cone of influence’. Colors indicate power spectra with warm colors denoting high power.

Yellow-band disease and dark-spot syndrome exhibited significant two- to three-year cycles in average prevalence, whereas white-band disease showed significant three- to four-year cycles in disease prevalence (Figs 1, 2 and Supplementary Figs 3–5). Wavelet analyses also showed significant El Niño events at a frequency of 2.7–5.3 years, which cycled especially predictably from 2003–2014 (Supplementary Fig. 2a and b). Moderate to strong El Niño events occurred in 1997–1998, 2002–2003, and 2009–2010 (Fig. 1 and Supplementary Fig. 1). Most compelling was that most disease records showed significant common periodicities with the ENSO cycle, particularly in the latter half of the time-series (i.e., from 2005 to 2014; Fig. 3 and Supplementary Figs 6–8).

Significant common periodicities between white-band disease and ENSO indices were identified for both *Acropora* species every three to four years (Fig. 3 and Supplementary Figs 6–8). For white-band disease on the threatened species *Acropora palmata*, significant three-year periodicities were detected, with disease peaking in 1999, 2004, 2006–2007, and 2009. Three of those four peaks occurred during weak or moderate El Niño events (Fig. 1). The temporal cycles of white-band disease on the threatened species *Acropora cervicornis* were less frequent than on *Acropora palmata*, with peaks in average disease prevalence occurring approximately every 4 years, in 1999, 2003, and 2008, with a particularly high disease prevalence in 2008 (~35%) (Supplementary Fig. 1). These results suggest that every three to four years, especially after 2005, environmental conditions associated with ENSO, including SST anomalies, may have driven these disease fluctuations (Supplementary Figs 1 and 6–8).

A two- to three-year frequency in the average prevalence of yellow-band disease was detected on the threatened corals *Orbicella faveolata* and *Orbicella franksi*. This periodicity was evident only from 1997–2005, and was clearly absent after the 2005 mass-bleaching event (Supplementary Figs 1 and 3–5), with the next peak in yellow-band disease on *Orbicella faveolata* delayed until 2012 (Fig. 1). After 2005, the average prevalence of the disease on *Orbicella franksi* did not exceed 1% (Supplementary Fig. 1). There also was a significant common periodicity between the Southern Oscillation Index and the maximum disease prevalence (Fig. 3 and Supplementary Fig. 7), suggesting a link between environmental conditions driven by climate cycles and disease outbreaks. However, the cross-wavelet signal was weak, which may be a consequence of the lack of new infections following the mass-mortality event in 2005–2006. Yellow-band disease is purportedly caused by a *Vibrio* bacterial pathogen^4^, and the disease is known to be influenced by sea-surface temperatures^19^.We hypothesize that the 2005 mass-bleaching event, which was followed by outbreaks of white-plague and yellow-band disease that resulted in high partial and whole-colony mortality in 2006 (refs 6, 7), may have removed the most susceptible genotypes from the population, delaying the next peak in disease prevalence by six years.

Dark-spot syndrome showed the strongest periodicity of any disease that was tested. A significant three-year cycle was detected in the average prevalence and in the maximum prevalence of dark-spot syndrome on *Siderastrea siderea* (Figs 1, 2, and Supplementary Figs 3–5). The prevalence of the disease peaked in 2003, 2006, 2009, 2012, and 2014, and was particularly high in 2009 (~13%) (Fig. 1). There were also significant three-year common periodicities in ENSO and dark-spot syndrome cycles (Fig. 3 and Supplementary Figs 5–7). While it is unknown whether dark-spot syndrome is an infectious and transmissible disease^20^, the wavelet results suggest that the disease may be influenced by environmental conditions associated with ENSO.

Overall, no long-term increases in disease prevalence were detected over the 18-year period, from 1997–2014 (Table 1), except for dark-spot syndrome, which significantly increased over time (0.4% per year, Table 1). These recent data suggest that the average prevalence of the major coral diseases in the Caribbean has not significantly changed over the last 18 years, supporting the findings of Ward and Lafferty^12^, and others^13, 14^. However, our temporal analyses are limited to the start of the regional monitoring program some 20 years ago, which did not capture the disease outbreaks that occurred during the late 1970s, throughout the 1980s, and into the early 1990s. These earlier outbreaks led to large decreases in host densities^21^ and decreases in disease-susceptible genotypes^22^. We hypothesize that these ecological changes, combined with increasing background sea-surface temperatures and short-term climate cycles have led to the present state of low background levels of disease punctuated with dynamic outbreaks. The significant coupling of coral-disease cycles with ENSO cycles, identified in this study, suggests that multi-year fluctuations in climate-driven environmental conditions influence the contemporary patterns of coral diseases in the Caribbean.

**Table 1:** Results of regression models evaluating long-term trends in the average annual prevalence (%) of three coral diseases in the Caribbean from 1997–2014.

Disease	Species	Slope	F-statistic	df	t-value	p-value	Adjusted R^2^
White-band disease	*Acropora cervicornis*	−0.096	0.146	345	−0.381	0.703	−0.002
	*Acropora palmata*	0.037	0.613	548	0.783	0.434	−0.001
	*Acropora* spp.	−0.034	0.109	789	−0.330	0.741	−0.001
Yellow-band disease	*Orbicella faveolata*	0.046	1.214	1229	1.102	0.271	0.000
	*Orbicella franksi*	−0.102	2.516	575	−1.586	0.113	0.003
	*Orbicella* spp.	0.024	0.795	1534	0.892	0.373	−0.000
Dark-spot syndrome	*Siderastrea siderea*	0.435	43.500	1303	6.595	**<0.001**	0.032

df denotes degrees of freedom.

**Figure 4:**
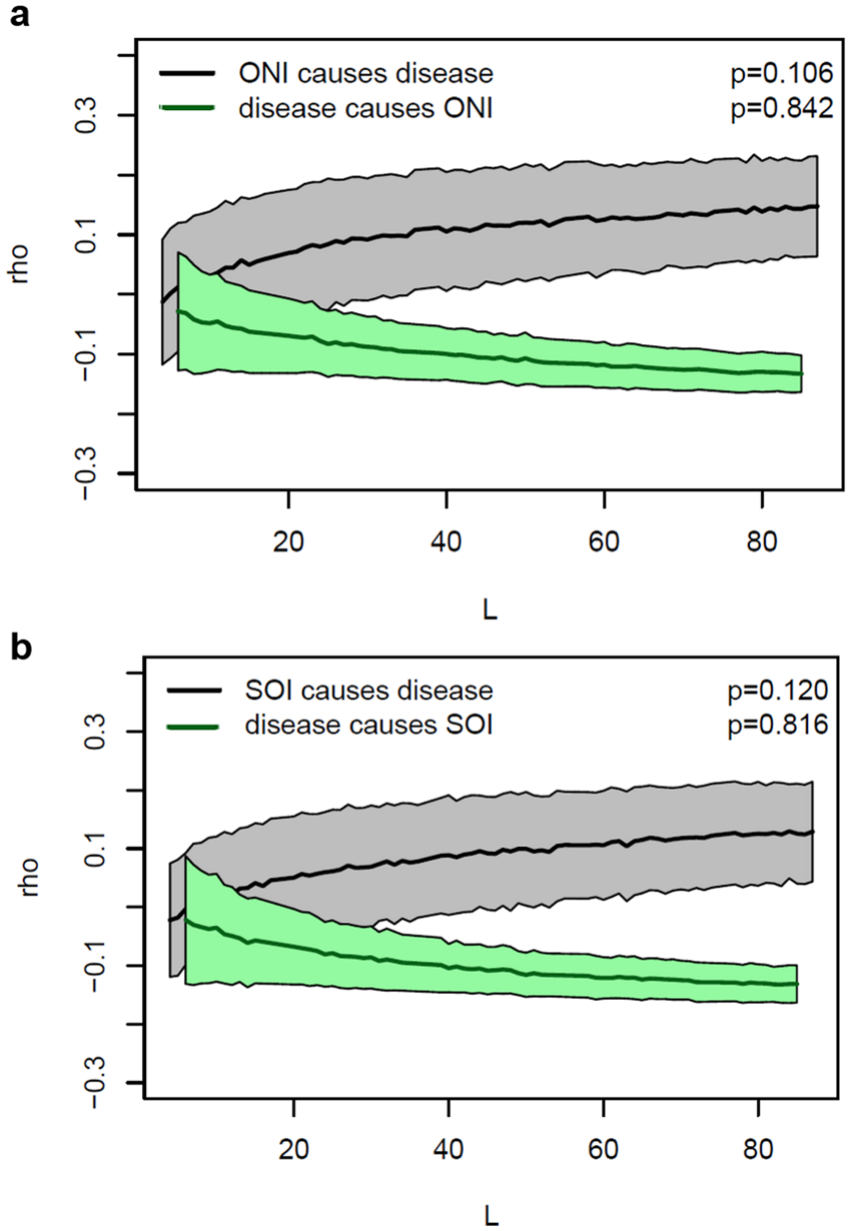
Multispatial convergent cross-mapping analyses. Lines and shaded regions indicate the mean ± 1 SD from 1000 bootstrapped model iterations. Causal forcing is indicated when the Pearson correlation coefficient (ρ) is greater than zero, for a large library length (L). Statistical significance (p) was used to determine whether the Pearson correlation coefficients for each process (ENSO and disease) were significantly greater than zero. (**a**) Oceanic Niño Index (ONI) and the prevalence of all coral diseases. (**b**) Southern Oscillation Index (SOI) and the prevalence of all coral diseases.

When all three diseases, on all five host species, were considered together, convergent cross-mapping (CCM) analyses indicated that conditions associated with the ONI and the SOI are likely drivers of the disease (Fig. 4). Despite the inherent variability in the prevalence data, and the small sample size of replicated time series, the CCM models consistently indicated that there was less than a 10% chance of obtaining the observed data, if no real causal effect existed (Fig. 4). The identification of large-scale climatic drivers of disease is not without precedent however. In terrestrial environments, the vector-borne protozoal disease cutaneous leishmaniasis peaks seasonally, but also cycles with a periodicity of 3.2 years in association with ENSO cycles^23^. Similarly, many tropical mosquito-borne diseases, including dengue fever^24^ and malaria^25^, synchronize with ENSO. This synchrony results from increasing temperatures and increasing rainfall during El Niño years, both of which increase mosquito populations, and consequently increase the rate of infectivity in human populations.

Clearly, local environmental conditions and population dynamics also influence patterns of disease prevalence, and this study does not suggest otherwise. Indeed, locally elevated nutrient levels have been shown to influence outbreaks of black-band disease^26^, and density-dependent processes have influenced outbreaks of white syndrome of the Great Barrier Reef ^21^. These local-scale and population-level processes likely contributed to the intra-annual variability in disease prevalence that was detected in this study (Supplementary Fig. 1). However, steady and low population densities across the survey sites, after the 1998 bleaching and mortality event (Supplementary Fig. 11), suggest that the patterns of disease prevalence that we detected were not driven by fluctuations in the density of the host populations.

The results of the present study suggest that large-scale climate signals, including ENSO indices, may be useful predictors of regional coral-disease outbreaks, defined as a marked increase in the incidence and prevalence of disease above background levels. Increasing greenhouse-gas emissions are expected to increase the frequency of strong El Niño events from one in every 20 years, to one in every 10 years^27^. Yet, perhaps even more notable is the gradual increase in sea-surface temperature that progressively raises the modal temperature thresholds of each ENSO cycle^28^. As a consequence of this rising background temperature, outbreaks of coral diseases may become more dynamic as the oceans continue to warm.

## Methods

### Coral-disease data

Data on coral diseases from 2082 sites throughout the Caribbean were obtained from the Atlantic and Gulf Rapid Reef Assessment (AGRRA) Program database, spanning from 1997 to 2014 (http://www.agrra.org/). At each survey site, the number of coral colonies of each species, and the number of coral colonies of each species with disease were recorded. Coral data were recorded from 1 m by 10 m belt transects, with an average of 6 ± 4 (SD) transects surveyed per site (minimum = 1, maximum = 31). The diseases of interest included: (1) white-band disease, (2) yellow-band disease, and (3) dark-spot syndrome, all of which had been described prior to the start of the surveys in 1997 (refs 29–31). We calculated site-level disease prevalence, and then averaged site-level data to get an estimate of yearly prevalence of each disease. We note that the distribution of surveys among months was similar across all years and for all diseases, and surveys were most frequent in August (Supplementary Fig. 9), although survey frequency varied somewhat among years (Supplementary Fig. 10). The maximum disease prevalence was also calculated for each year (Supplementary Fig. 1). The prevalence of white-band disease on both *Acropora* species in 2010 was interpolated as the average of the mean prevalence for 2009 and 2011, because no *Acropora* were surveyed in 2010, and missing data are problematic using wavelet analyses. Similarly, the prevalence of white-band disease on *A. cervicornis* in 2007 was interpolated. However, we also ran the models assuming a prevalence value of zero during the years with no data, and the wavelet results did not change.

### Environmental Data

The El Niño Southern Oscillation (ENSO) is a periodic climatic fluctuation in the trade winds and the sea-surface temperature across the tropical Pacific Ocean. Tropical Atlantic and Caribbean sea-surface temperatures are influenced by ENSO, whereby anomalously warm sea-surface temperatures in the Caribbean lag 1–5 months after the mature phase of the Pacific warm period^11^ (also see *Validation of Caribbean SST and ENSO relationship* below). The SOI is a measure of the difference in the air pressure between the western and eastern tropical Pacific Ocean. Negative pressure differentials indicate El Niño-like conditions, whereas positive pressure differentials indicate La Niña-like conditions. Monthly Southern Oscillation Index (SOI) values were obtained from the National Oceanic and Atmospheric Administration’s (NOAA) National Centers for Environmental Information (https://www.ncdc.noaa.gov/teleconnections/enso/indicators/soi/). The Oceanic Niño Index (ONI) was obtained from NOAA (http://www.cpc.ncep.noaa.gov/products/analysis_monitoring/ensostuff/ensoyears.shtml), which was calculated using the Extended Reconstructed Sea Surface Temperature (ERSST) v4 data set. The ONI is the 3-month running anomaly of sea-surface temperature for the Niño 3.4 region, located in the tropical Pacific at latitude 5°N–5°S and longitude 120°W–170°W. The ONI is another standard measure of the ENSO cycle. In the case of the ONI, positive anomalies indicate El Niño-like conditions, whereas negative anomalies indicate La Niña-like conditions. The centered, 30-year base period ‘ClimAdjust’ version of the ONI data were used for all calculations (http://www.cpc.ncep.noaa.gov/products/analysis_monitoring/ensostuff/ONI_change.shtml). Yearly averages of the SOI and the ONI were calculated and compared with yearly estimates of disease prevalence.

### Data analyses

The average yearly prevalence of each disease, on each host coral species, was calculated and the disease time-series was detrended to remove any potential linearity in the data, which would confound the wavelet analyses. Records were detrended in the statistical program R^32^. Morlet wavelet-transform analyses were then used to identify significant frequencies (i.e., periodicities) in the detrended records, and to identify the timing of those events. The wavelet power spectra were used as an indication of the strength of the signals in each disease record. The two indices of ENSO (i.e., the SOI and the ONI) also were detrended, and cross-wavelet analyses were used to identify significant common periodicities between disease prevalence and ENSO signals. Chi-square tests were used to determine whether a peak in the wavelet power spectrum was significantly above the background spectrum with a 95% confidence level. Wavelet analyses were conducted in the statistical program R^32^ (R Core Team, 2016) using the packages ‘dplR’^33^ and ‘biwavelet’^34^.

Convergent cross mapping was used to detect the presence and direction of causal relationships between both ENSO indices and the prevalence of each coral disease by comparing the time-lagged components of one process against the dynamics of the other process^35^. For this model, the time series of the prevalence of each disease, on each host species examined, was considered a replicate, for a total of five replicated time series. A five-step algorithm was used to develop the model, as described in ref. 35. Briefly, diagnostic plots were used to determine the best embedding dimension for the analysis, which set the number of time steps used for the prediction. Next, the embedding dimension was used to make predictions of the observations, increasingly into the future, to test for nonlinearity and stochastic noise. If the processes are non-linear, predictive power will decrease over time. Third, the abilities of each process (i.e., ENSO and disease) to predict each other process are tested using CCM. Bootstrapping, using 1000 iterations per test, then was used to leverage the replicate time series. Pearson correlation coefficients were calculated for each iteration, comparing the predicted estimates from the CCM model to the observed values. Finally, tests for statistical significance were used to determine whether the Pearson correlation coefficients for each process were significantly greater than zero. All CCM models were conducted in the statistical program, R^32, 35^, using the package ‘multispatialCCM’^36^. Generalized linear models were used to examine long-term trends in disease prevalence over the 18-year period in base R^32^.

### Validation of Caribbean SST and ENSO relationship

A study that evaluated the relationship between tropical Atlantic SST and ENSO behavior found that between 50 and 80% of the SST anomalies in the North Atlantic and Caribbean were associated with the Pacific ENSO^16^. This relationship is primarily driven by reductions in the north-east trade winds during El Niño periods that reduce heat loss over the tropical Atlantic, and tend to lag the ENSO cycle in the Pacific Ocean by 1–5 months^16^. To validate the link between tropical Atlantic SST and ENSO, basin-wide yearly SST anomaly records were compared with the ONI and the SOI (See *Environmental Data* above). Monthly nighttime sea-surface temperature records, at a 4 km by 4 km spatial resolution, were obtained from the Advanced Very High Resolution Radiometer (AVHRR) Pathfinder 5.2 (PFV5.2) (http://pathfinder.nodc.noaa.gov). Monthly SSTs were averaged to calculate annual mean SSTs for each 4 km by 4 km pixel, which were then averaged over a region encompassing the Caribbean Sea, the Gulf of Mexico and the South Atlantic Ocean to derive yearly averages in SST. This basin-wide annual mean SST record was detrended, centered, and compared with the two indices of ENSO using cross-wavelet analyses, as described above. Significant, common 4-year periodicities were detected in SST anomalies and both ENSO indices over most of the 31-year record, supporting the link between Caribbean SST and ENSO (Supplementary Fig. 12).

## Electronic supplementary material

Supplementary document

## References

[1] Rogers C (2009). Coral bleaching and disease should not be underestimated as causes of Caribbean coral reef decline. Proc. R. Soc. B.

[2] Harvell CD (1999). Emerging Marine Diseases–Climate Links and Anthropogenic Factors. Science.

[3] Randall CJ, van Woesik R (2015). Contemporary white-band disease in Caribbean corals driven by climate change. Nature Climate Change.

[4] Cervino JM (2004). Relationship of *Vibrio* Species Infection and Elevated Temperatures to Yellow Blotch/Band Disease in Caribbean Corals. Appl. Environ. Microbiol..

[5] Gil-Agudelo DL, Garzón-Ferreira J (2001). Spatial and seasonal variation of dark spots disease in coral communities of the Santa Marta area (Colombian Caribbean). Bull. Mar. Sci..

[6] Cróquer A, Weil E (2009). Changes in Caribbean coral disease prevalence after the 2005 bleaching event. Dis. Aquat. Org..

[7] Miller J (2009). Coral disease following massive bleaching in 2005 causes 60% decline in coral cover on reefs in the US Virgin Islands. Coral Reefs.

[8] Rogers, C. S., Miller, J., & Muller, E. M. Coral diseases following massive bleaching in 2005 cause 60 percent decline in coral cover and mortality of the threatened species, *Acropora palmata*, on reefs in the US Virgin Islands. *USGS Fact Sheet*, *3058* (2008).

[9] Brandt ME, McManus JW (2009). Disease incidence is related to bleaching extent in reef‐building corals. Ecology.

[10] Precht WF, Gintert BE, Robbart ML, Fura R, van Woesik R (2016). Unprecedented disease-related coral mortality in Southeastern Florida. Scientific Reports.

[11] Maynard J (2016). Improving marine disease surveillance through sea temperature monitoring, outlooks and projections. Phil. Trans. R. Soc. B..

[12] Ward JR, Lafferty KD (2004). The elusive baseline of marine disease: are diseases in ocean ecosystems increasing?. PLoS Biol..

[13] Bruno JF, Ellner SP, Vu I, Kim K, Harvell CD (2011). Impacts of aspergillosis on sea fan coral demography: modeling a moving target. Ecological Monographs.

[14] Harvell D, Altizer S, Cattadori IM, Harrington L, Weil E (2009). Climate change and wildlife diseases: when does the host matter the most?. Ecology.

[15] Altizer S (2006). Seasonality and the dynamics of infectious diseases. Ecol. Lett.

[16] Enfield DB, Mayer DA (1997). Tropical Atlantic sea surface temperature variability and its relation to El Niño-Southern Oscillation. J. Geophys. Res.: Oceans.

[17] Weil, E., & Rogers, C. S. Coral Reef Diseases in the Atlantic-Caribbean in *Coral Reefs: An Ecosystem in Transition* (eds. Dubinsky, Z. & Stambler, N.) 465–491 (Springer, 2011).

[18] Aronson RB, Precht WF (2001). White-band disease and the changing face of Caribbean coral reefs. Hydrobiologia.

[19] Cervino JM (2008). The *Vibrio* core group induces yellow band disease in Caribbean and Indo-Pacific reef-building corals. J. Appl. Microbiol..

[20] Randall CJ, Jordán-Garza AG, Muller EM, Van Woesik R (2016). Does dark-spot syndrome experimentally transmit among Caribbean Corals?. PLoS ONE.

[21] Bruno J (2007). Thermal stress and coral cover as drivers of coral disease outbreaks. PLoS Biol.

[22] Vollmer SV, Kline DI (2008). Natural disease resistance in threatened staghorn corals. Plos one.

[23] Franke CR, Ziller M, Staubach C, Latif M (2002). Impact of the El Niño/Southern Oscillation on Visceral Leishmaniasis, Brazil. Emerging Infectious Diseases.

[24] Cazelles B, Chavez M, McMichael AJ, Hales S (2005). Nonstationary influence of El Niño on the synchronous dengue epidemics in Thailand. PLoS Med.

[25] Zhou G, Minakawa N, Githeko AK, Yan G (2004). Association between climate variability and malaria epidemics in the East African highlands in. Proc. Nat. Acad. Sci. USA.

[26] Kuta K, Richardson L (2002). Ecological aspects of black band disease of corals: relationships between disease incidence and environmental factors. Coral Reefs.

[27] Cai W (2014). Increasing frequency of extreme El Niño events due to greenhouse warming. Nature Climate Change.

[28] Rhein, M. *et al*. Observations: Ocean in *Climate Change 2013 The Physical Science Basis in Working Group I Contribution to the Fifth Assessment Report of the Intergovernmental Panel on Climate Change* (eds Stocker, T. F. *et al*.) 255–316 (Cambridge University Press 2013).

[29] Gladfelter WB (1982). White-band disease in *Acropora palmata*: implications for the structure and growth of shallow reefs. Bull. Mar. Sci..

[30] Garzón-Ferreira J, Gil DL (1998). Another unknown Caribbean coral phenomenon. Reef Encounter.

[31] Goreau TJ (1998). Rapid Spread of Diseases in Caribbean Coral Reefs. Revista de Biología Tropical.

[32] R Core Team. R: A language and environment for statistical computing. R Foundation for Statistical Computing, Vienna, Austria. http://www.R-project.org (2016).

[33] Bunn AG (2008). A dendrochronology program library in R (dplR). Dendrochronologia.

[34] Gouhier, T. Biwavelet: Conduct univariate and bivariate wavelet analyses (Version 0.17.4). http://github.com/tgouhier/biwavelet (2014).

[35] Clark TC (2015). Spatial convergent cross mapping to detect causal relationships from short time series. Ecology.

[36] Clark, T. C. MultispatialCCM: Multispatial Convergent Cross Mapping (R package version 1.0). http://CRAN.R-project.org/package=multispatialCCM (2015).

