# Author Correction: Some coral diseases track climate oscillations in the Caribbean

**DOI:** 10.1038/s41598-018-23087-x

**Published:** 2018-03-19

**Authors:** C. J. Randall, R. van Woesik

**Affiliations:** Department of Biological Sciences, Florida Institute of Technology Melbourne, Florida, United States of America

Correction to: *Scientific Reports* 10.1038/s41598-017-05763-6, published online 18 July 2017

The Acknowledgements section in this Article is incomplete:

“We thank the Atlantic and Gulf Rapid Reef Assessment Program, especially K. Marks, J. Lang, and P. Kramer for providing data. Thanks also to S. J. van Woesik for editorial comments. This paper is Contribution No. 148 from the Institute for Research on Global Climate Change at the Florida Institute of Technology.”

should read:

“We thank the Atlantic and Gulf Rapid Reef Assessment Program, especially K. Marks, J. Lang, and P. Kramer for providing data. Thanks also to S. J. van Woesik for editorial comments. This paper is Contribution No. 148 from the Institute for Research on Global Climate Change at the Florida Institute of Technology. CJR was supported by a National Science Foundation (NSF) OCE grant 1535007 to Richard B. Aronson. RvW acknowledges funding from the National Science Foundation (NSF) OCE grant 1657633.”

