# Supplementary material for: Some coral diseases track climate oscillations in the Caribbean
Source: Sci Rep. 2017 Jul 18;7:5719. doi: 10.1038/s41598-017-05763-6 (PMC5515922; doi:10.1038/s41598-017-05763-6)
Supplement: Supplementary file 1 — Supplementary document [file 41598_2017_5763_MOESM1_ESM.doc]

**Supplementary Document:**

**Some coral diseases track climate oscillations in the Caribbean**

C. J. Randall^*^ and R. van Woesik

Department of Biological Sciences

Florida Institute of Technology

Melbourne, Florida

United States of America

^*^Corresponding author

Email: [crandall2012@my.fit.edu](mailto:crandall2012@my.fit.edu)

**Supplementary Figures**

**Figure S1.** Yearly average prevalence (%) and maximum prevalence (%) of three coral diseases on scleractinian coral species in the Caribbean from 1997–2014. **a**. White-band disease on *Acropora cervicoris*. **b**. White-band disease on *Acropora palmata*. **c**. Yellow-band disease on *Orbicella faveolata*. **d**. Yellow-band disease on *Orbicella franksi*. **e**. Dark-spot syndrome on *Siderea siderea.* Error bars represent ± 1 standard error of the mean. Values above the points indicate the sample size (i.e. the number of surveys) used for the calculations of average disease prevalence. Shaded areas indicate the strength of the El Niño periods: Dark gray indicates very strong El Niño 1.5–1.9 °C sea-surface temperature (SST) anomaly, gray indicates moderate El Niño periods 1.0–1.4 °C SST anomaly, and light gray indicates weak El Niño periods 0.5–0.9 °C SST anomaly.

**Figure S2.** Morlet wavelet-transform analyses of values of monthly El Niño Southern Oscillation indices from 1997–2015 (**a** and **b**) and from 1950–2015 (**c** and **d**). Oceanic Niño Index (ONI; **a** and **c**). Southern Oscillation Index (SOI; **b** and **d**). Regions within black contour lines indicate statistically significant periodicites at p<0.05. Dashed areas indicate the region outside the ‘cone of influence’. Color scales indicate the power spectra.

**Figure S3.** Morlet wavelet-transform analyses of the average yearly prevalence of three coral diseases on scleractinian coral species in the Caribbean from 1997–2015. **a**. White-band disease on *Acropora cervicoris*. **b**. White-band disease on *Acropora palmata*. **c**. Yellow-band disease on *Orbicella faveolata*. **d**. Yellow-band disease on *Orbicella franksi*. **e**. Dark-spot syndrome on *Siderea siderea.* Regions within black contour lines indicate statistically significant periodicites at p<0.05. Dashed areas indicate the region outside the ‘cone of influence’. Color scales indicate the power spectra.

**Figure S4.** Morlet wavelet-transform analyses of the maximum yearly prevalence of three coral diseases on scleractinian coral species in the Caribbean from 1997–2015. **a**. White-band disease on *Acropora cervicoris*. **b**. White-band disease on *Acropora palmata*. **c**. Yellow-band disease on *Orbicella faveolata*. **d**. Yellow-band disease on *Orbicella franksi*. **e**. Dark-spot syndrome on *Siderea siderea.* Regions within black contour lines indicate statistically significant periodicites at p<0.05. Dashed areas indicate the region outside the ‘cone of influence’. Color scales indicate the power spectra.

**Figure S5.** Morlet wavelet-transform analyses of the variance in yearly prevalence of three coral diseases on scleractinian coral species in the Caribbean from 1997–2015. **a**. White-band disease on *Acropora cervicoris*. **b**. White-band disease on *Acropora palmata*. **c**. Yellow-band disease on *Orbicella faveolata*. **d**. Yellow-band disease on *Orbicella franksi*. **e**. Dark-spot syndrome on *Siderea siderea.* Regions within black contour lines indicate statistically significant periodicites at p < 0.05. Dashed areas indicate the region outside the ‘cone of influence’. Color scales indicate the power spectra.

**Figure S6.** Cross-wavelet analyses comparing yearly average prevalence of three coral diseases on scleractinian coral species in the Caribbean with The Southern Oscillation Index (SOI). Regions within black contour lines indicate statistically significant periodicites at p<0.05. Dashed areas indicate the region outside the ‘cone of influence’. Color scales indicate the power spectra.

**Figure S7.** Cross-wavelet analyses comparing yearly maximum prevalence of three coral diseases on scleractinian coral species in the Caribbean with the Southern Oscillation Index (SOI). Regions within black contour lines indicate statistically significant periodicites at p<0.05. Dashed areas indicate the region outside the ‘cone of influence’. Color scales indicate the power spectra.

**Figure S8.** Cross-wavelet analyses comparing the variance in yearly average prevalence of four coral diseases on scleractinian coral species in the Caribbean with the Southern Oscillation Index (SOI). Regions within black contour lines indicate statistically significant periodicites at p<0.05. Dashed areas indicate the region outside the ‘cone of influence’. Color scales indicate the power spectra.

**Figure S9.** Frequency distributions of surveys of each coral species from all years, over the 12 months of the year, beginning with January (1) and ending with December (12).

**Figure S10.** Frequency distributions indicating the number of surveys performed each year, and from each season within eacbh year.

**Figure S11**. Average yearly colony density (# colonies per 10 m^2^ transect) of each coral species through time.

**Figure S12.** Cross-wavelet analyses comparing the basin-wide yearly sea-surface temperature (SST) anomaly record with the Oceanic Nino Index (ONI; left) and the Southern Oscillation Index (SOI; right) from 1982-2012. Regions within black contour lines indicate statistically significant periodicites at p<0.05. Dashed areas indicate the region outside the ‘cone of influence’. Color scales indicate the power spectra.


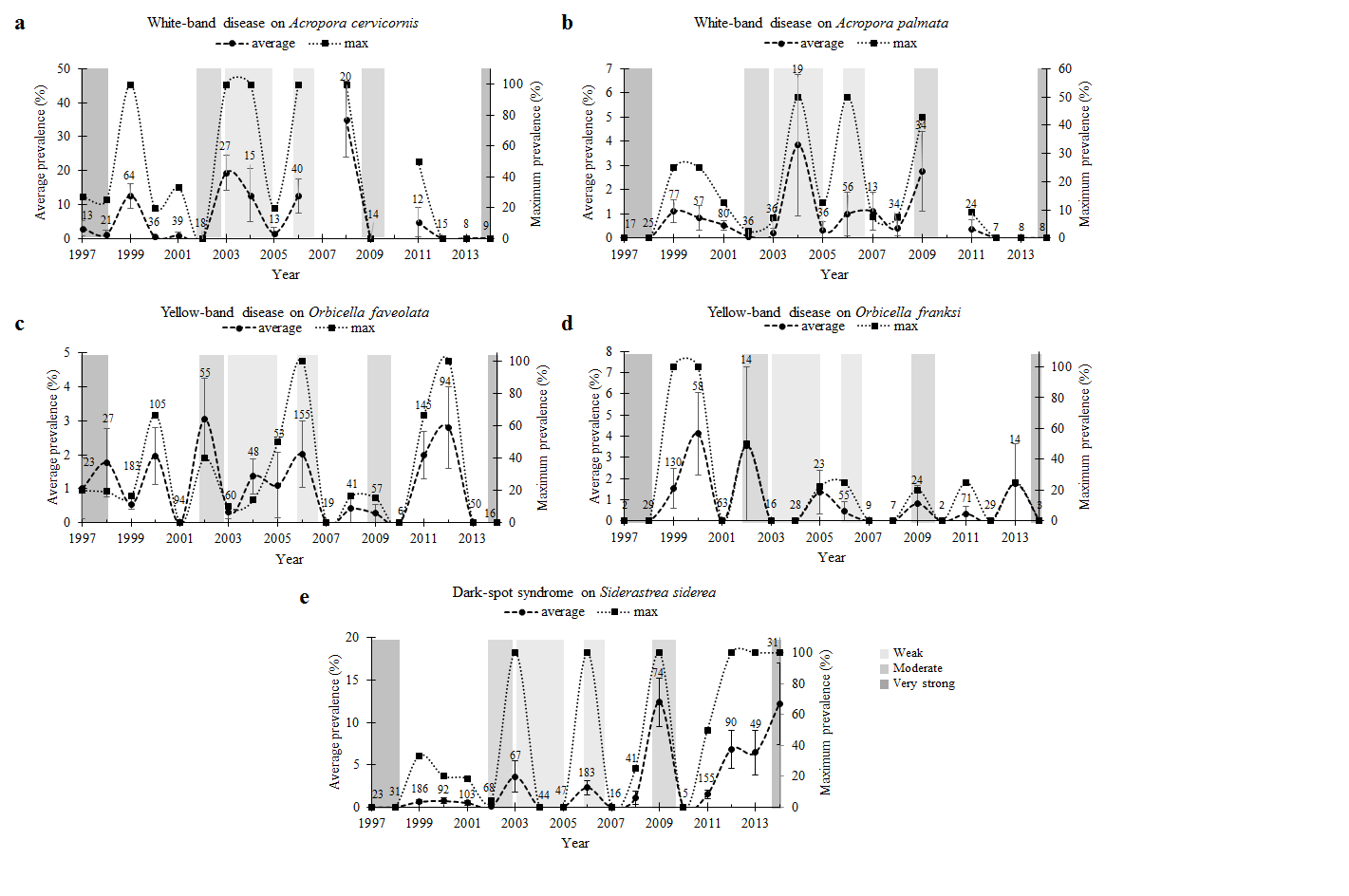


**Supplementary Figure 1.**


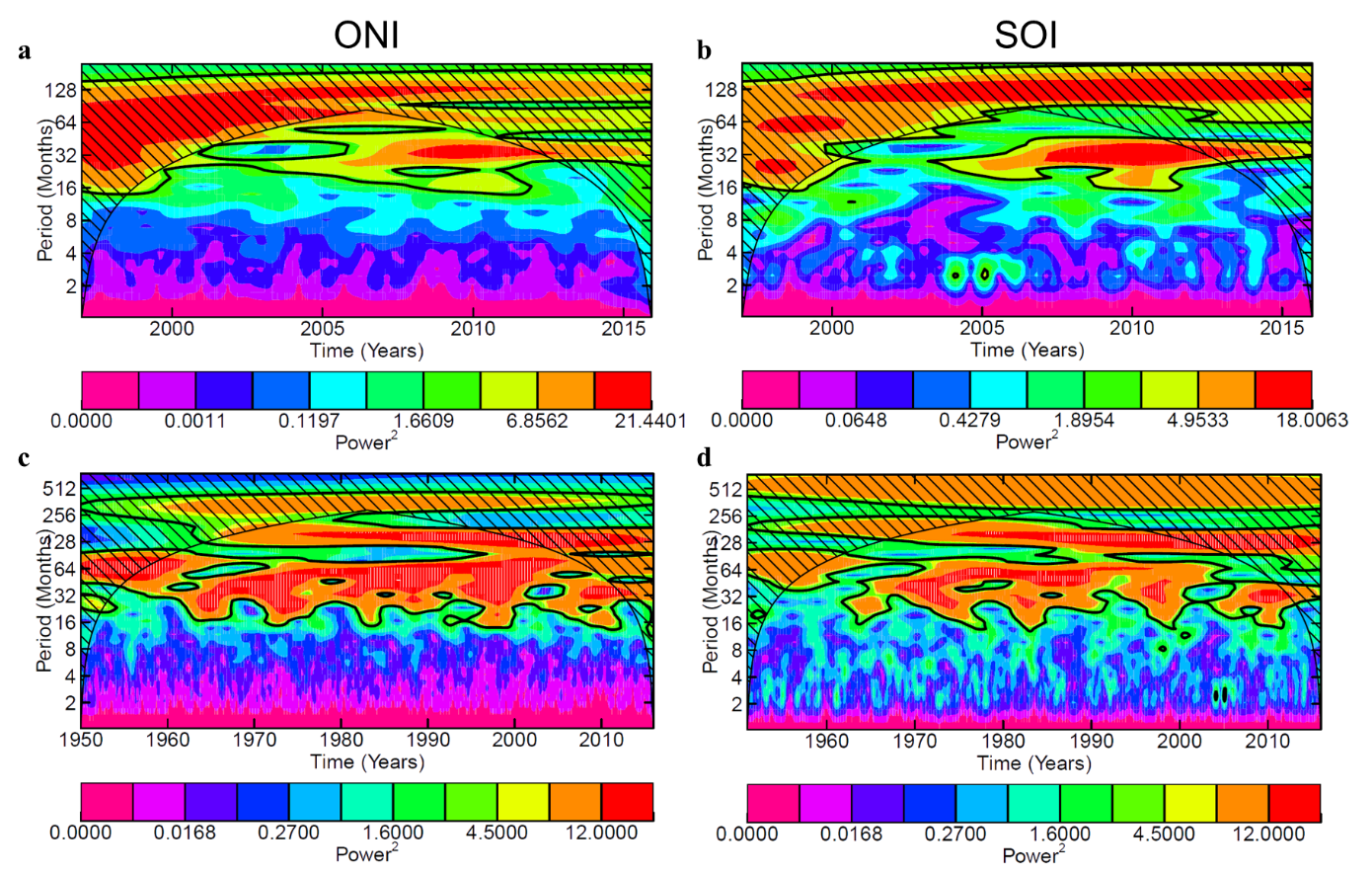


**Supplementary Figure 2.**

**
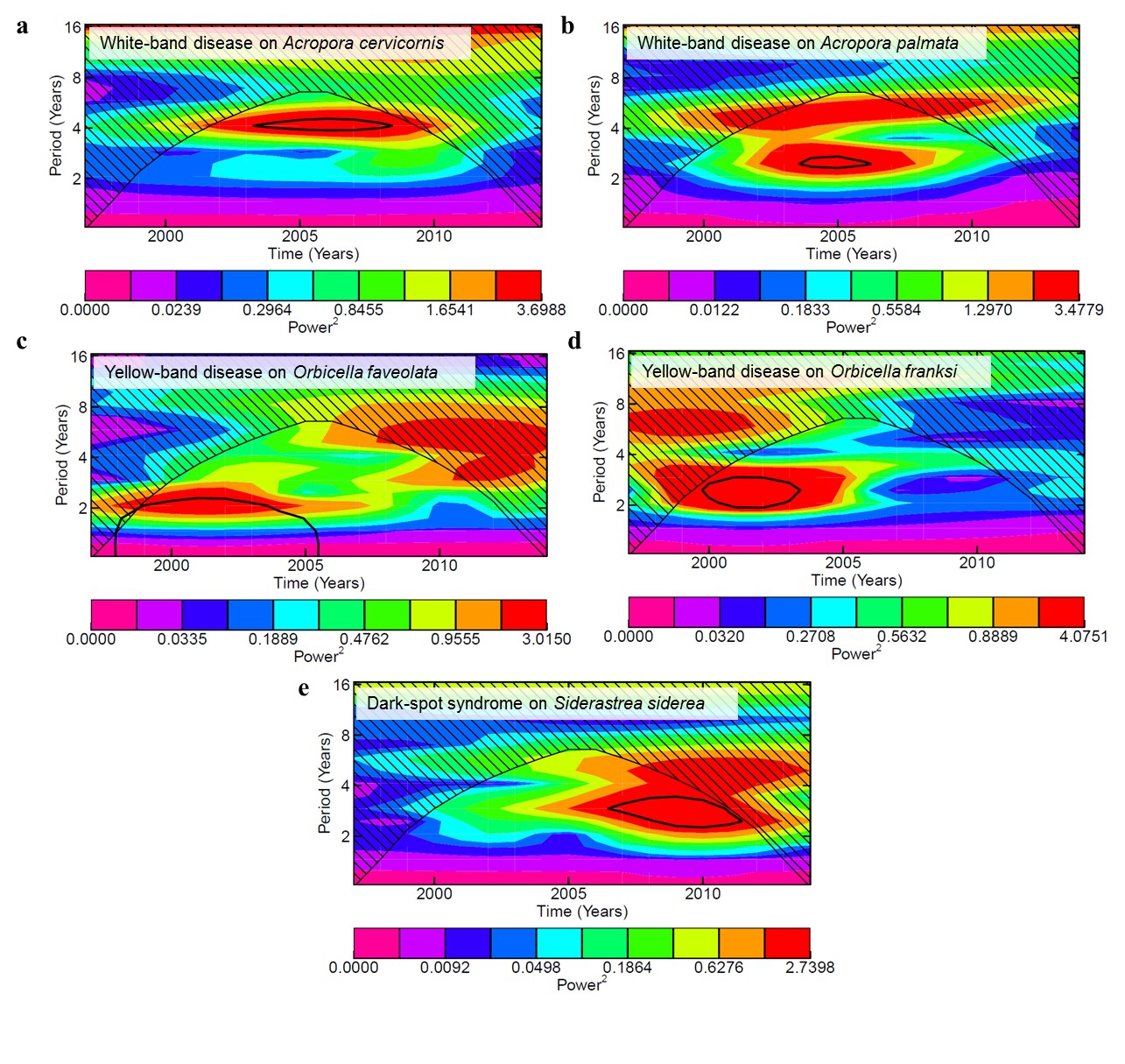
**

**Supplementary Figure 3.**


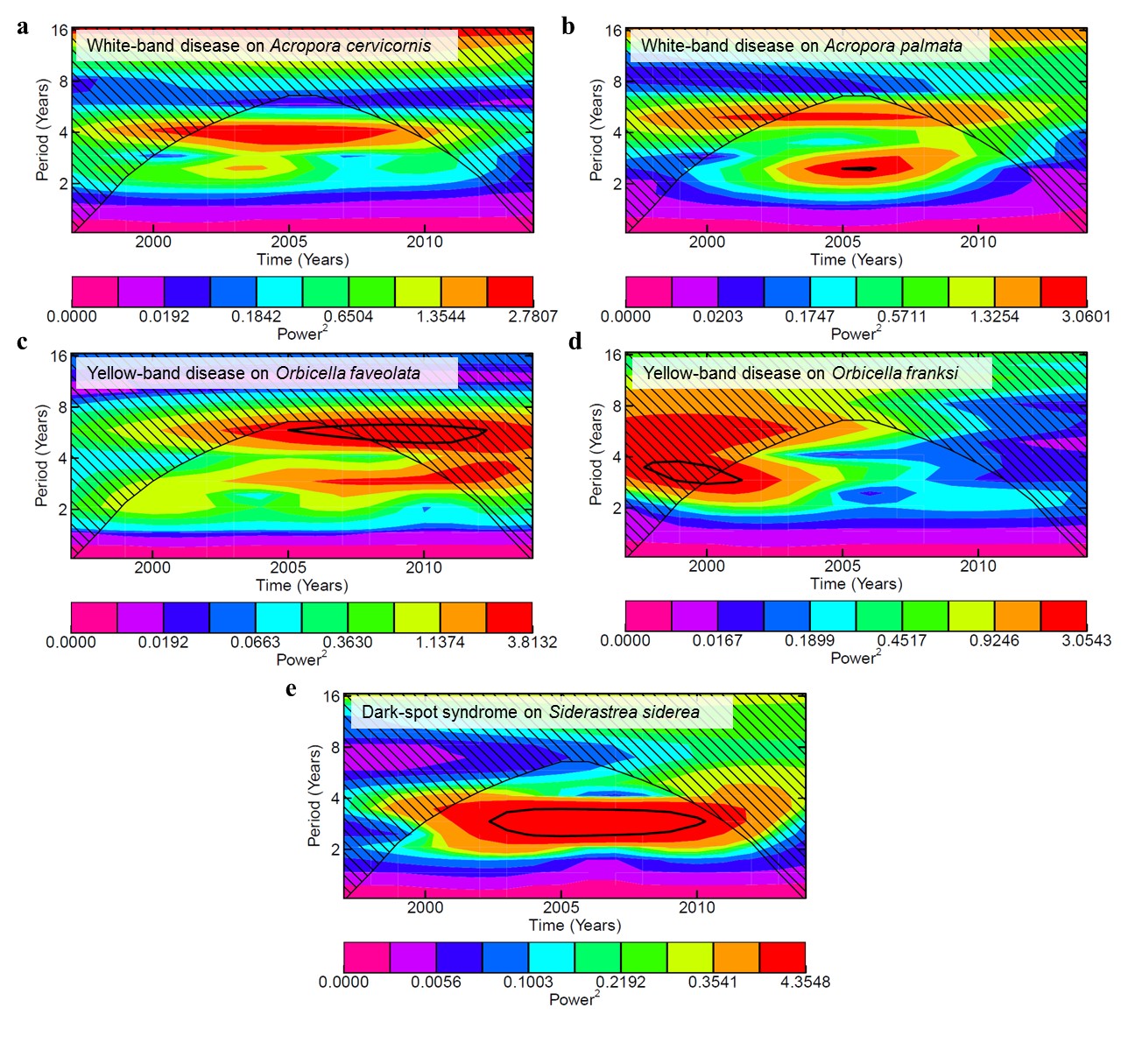


**Supplementary Figure 4.**

**
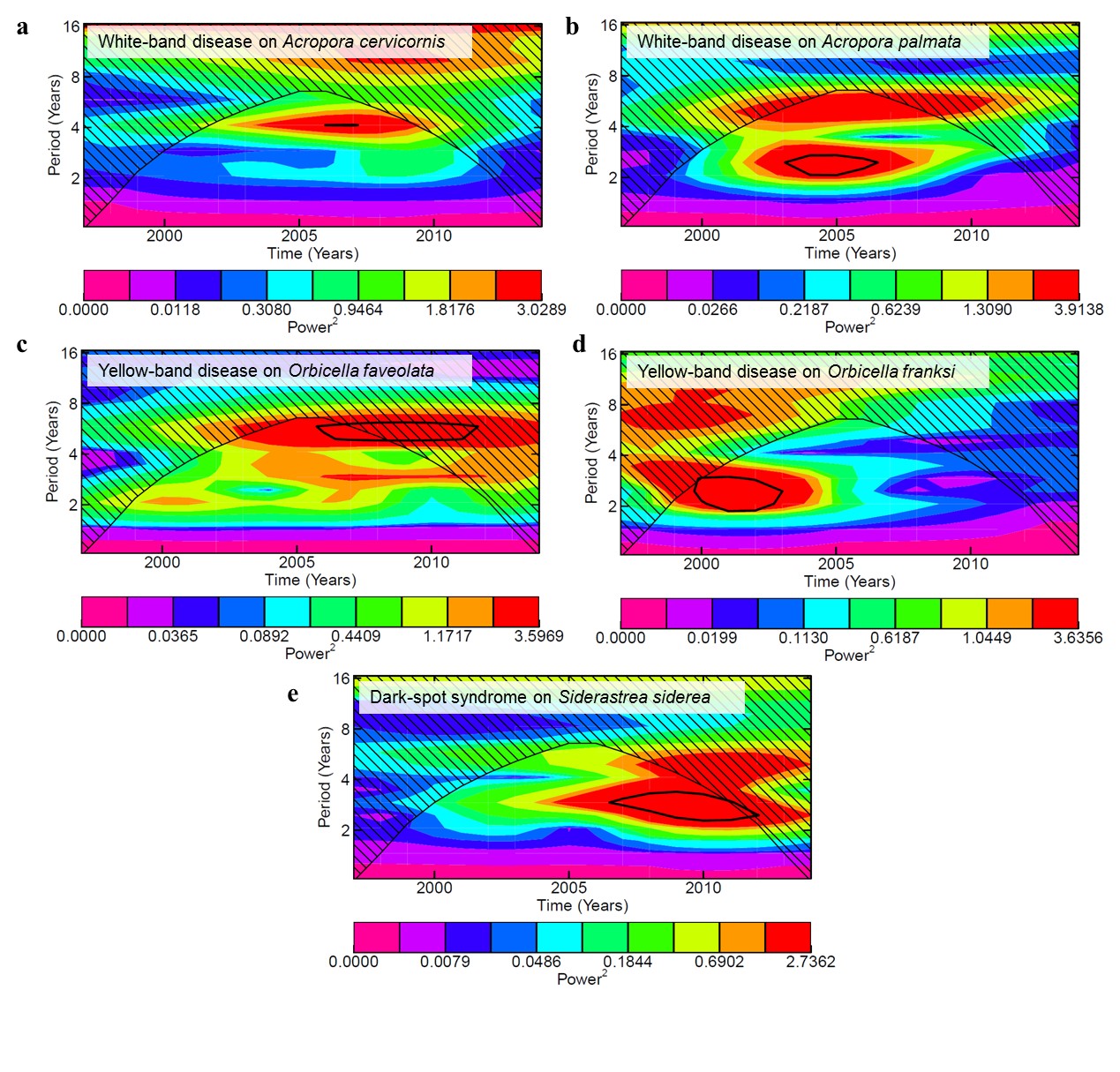
**

**Supplementary Figure 5.**


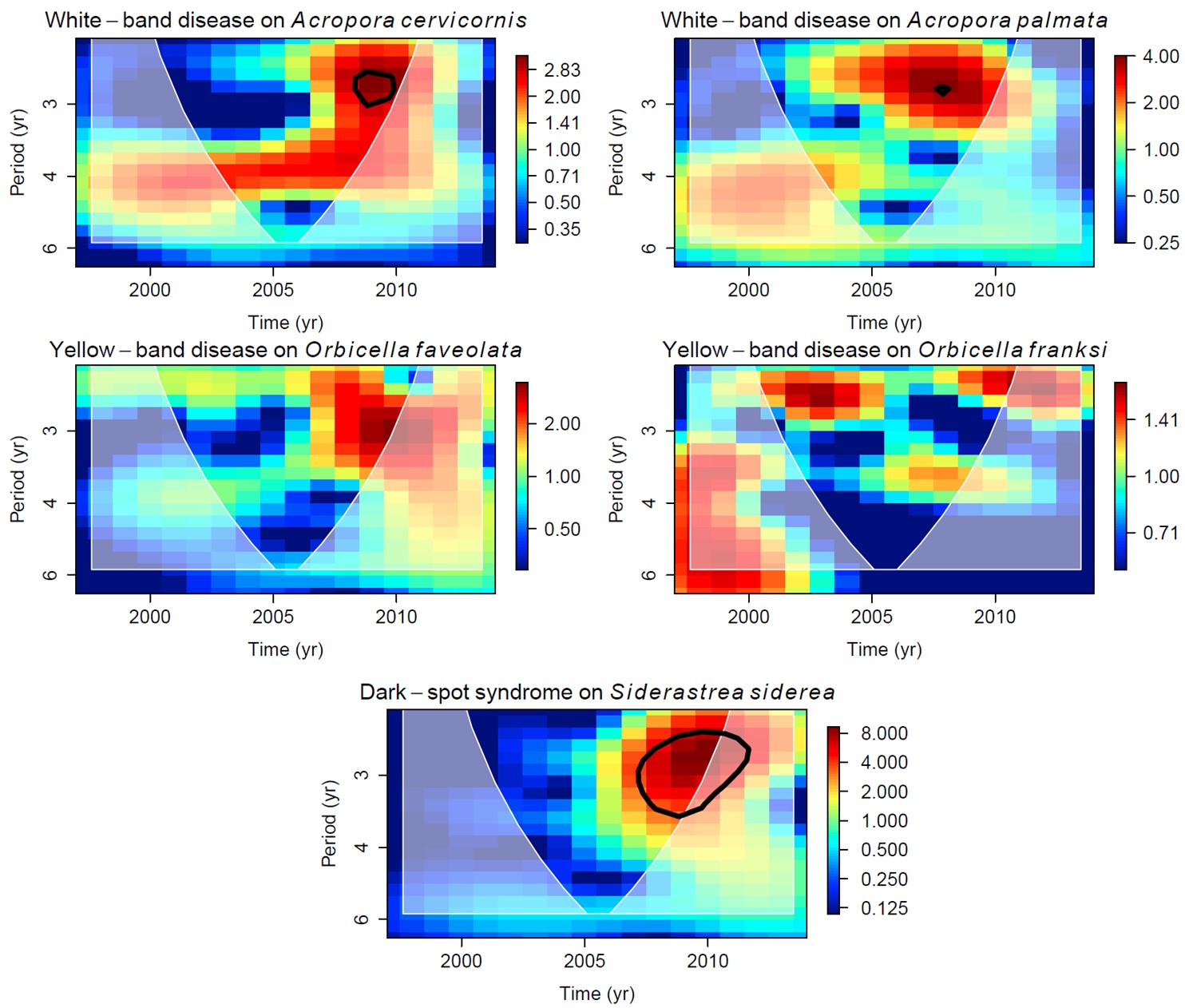


**Supplementary Figure 6.**


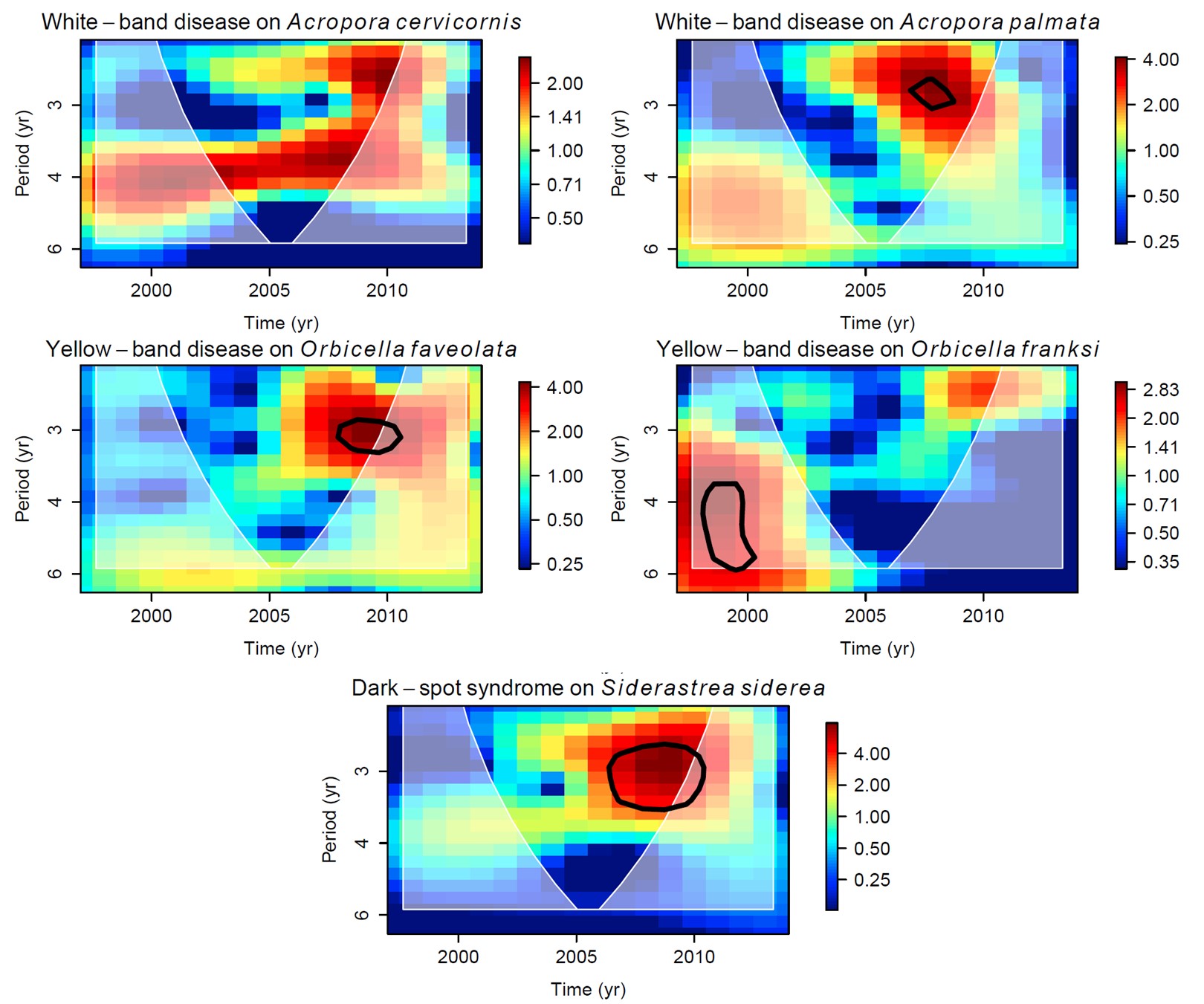


**Supplementary Figure 7.**

**
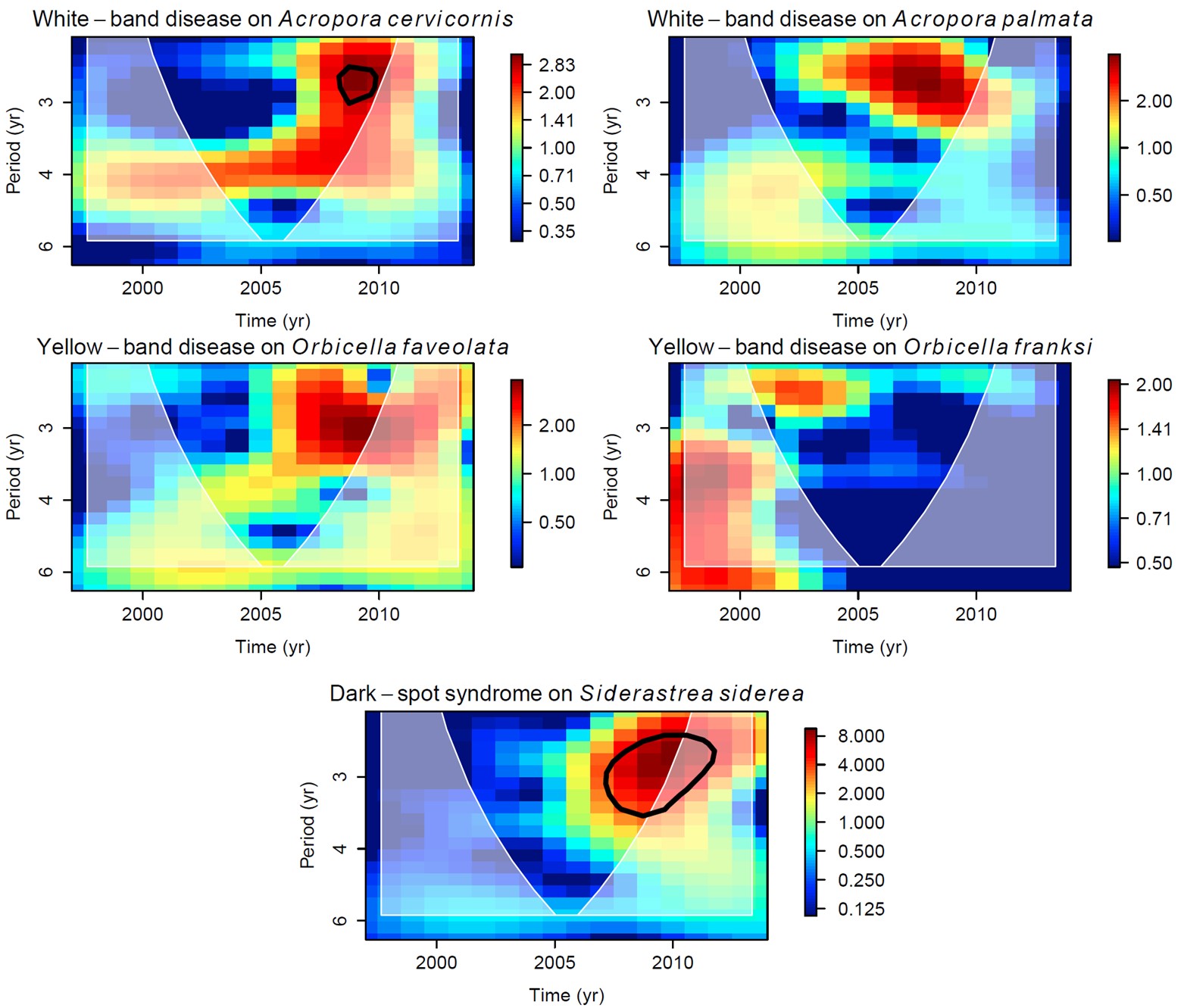
**

**Supplementary Figure 8.**

**
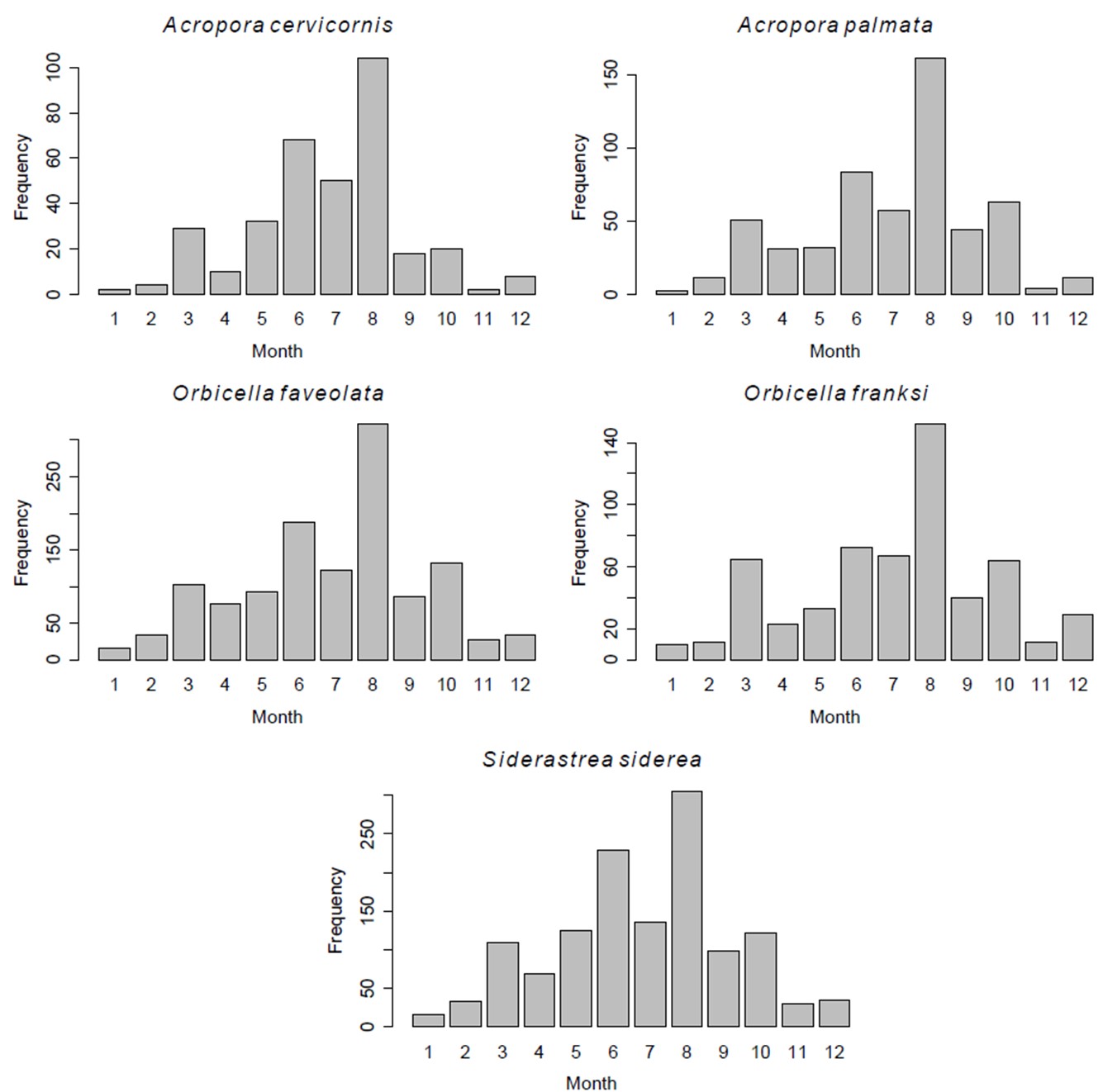
**

**Supplementary Figure 9.**


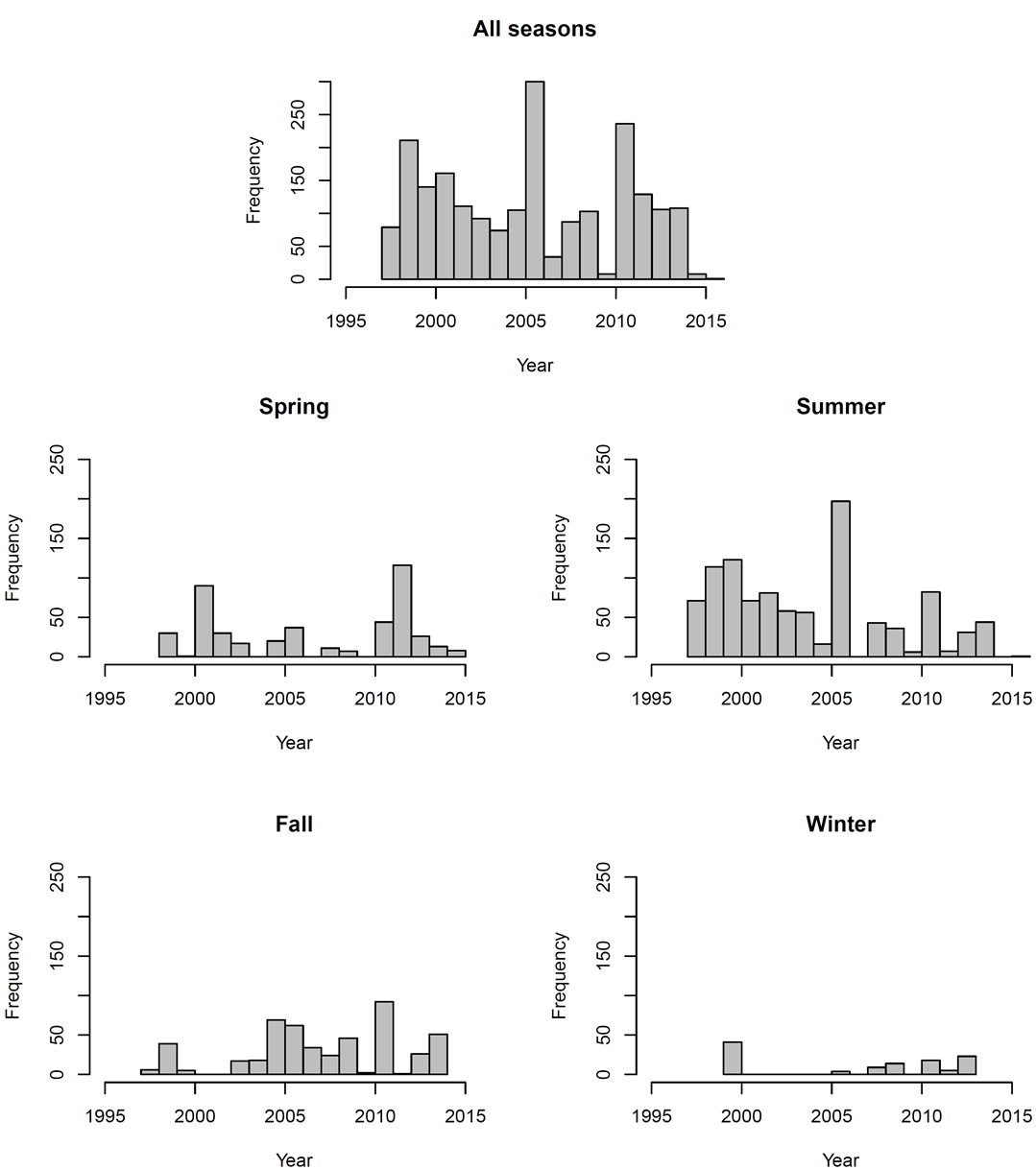


S**upplementary Figure 10.**


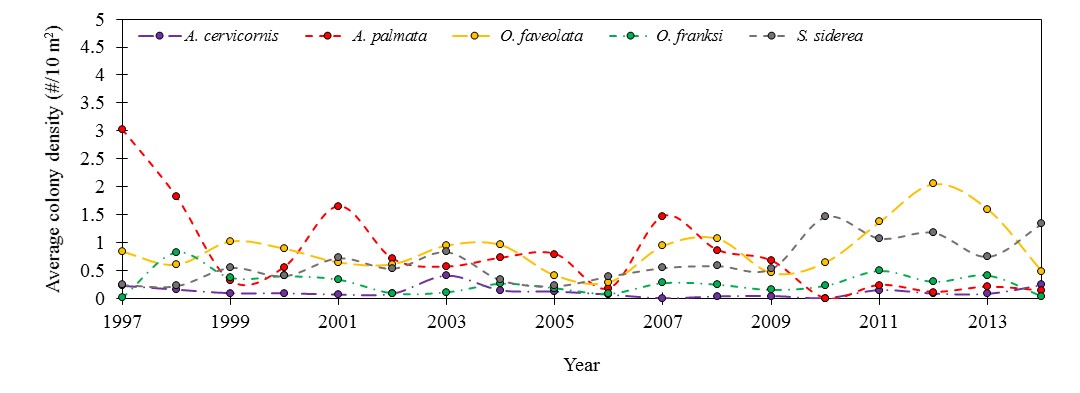


**Supplementary Figure 11**.


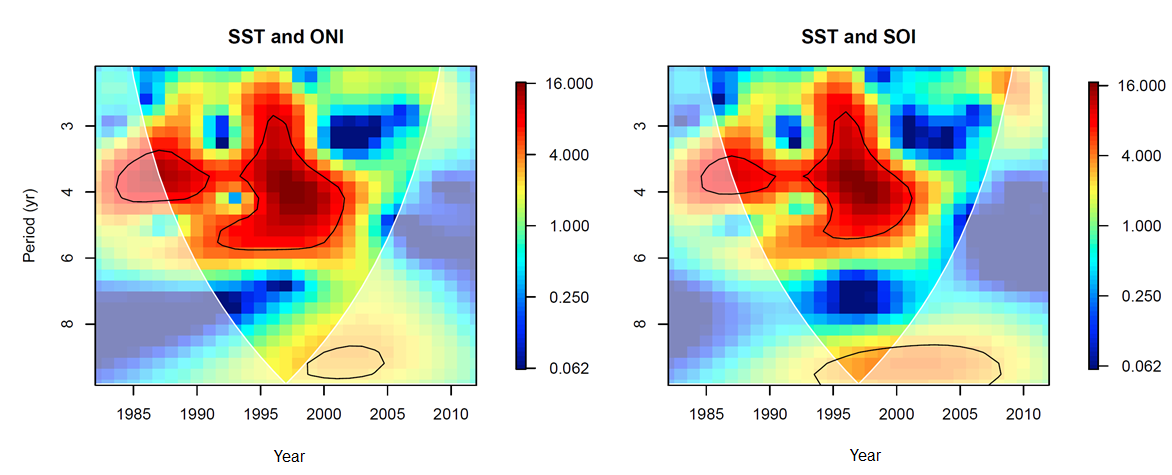


**Supplementary Figure 12**.
